# The profunda artery perforator flap for upper limb reconstruction: A case report and literature review on the flap applications in reconstruction

**DOI:** 10.1002/micr.30941

**Published:** 2022-07-25

**Authors:** Filippo Boriani, Paolo Sassu, Matteo Atzeni, Christina Buckley, Andrea Figus

**Affiliations:** ^1^ Department of Plastic Surgery and Microsurgery University of Cagliari Cagliari Italy; ^2^ Department of Orthoplastics IRCCS, Istituto Ortopedico Rizzoli Bologna BO 40136 Italy

## Abstract

The profunda femoris artery perforator (PAP) flap has been recently popularized as an alternative option for microsurgical reconstruction. The use of PAP flap has never been reported and described for reconstruction of the upper extremities, in particular the forearm. The purpose of this case report is to describe a case suggesting the PAP flap as a further reconstructive option in the upper limb. A 16‐year‐old girl who sustained a traumatic injury to her right dominant forearm resulting in subtotal circumferential tissue loss following a road traffic accident was referred to the authors' department 2 years post‐trauma. The disabling fibrotic sequelae on her volar forearm (15 × 10 cm) resulted in a nonfunctional hand. She was unable to perform any active movement of her wrist or digits. Passive movements in the finger joints were preserved. Following debridement and reconstruction of nerves and tendons, soft tissues were resurfaced with a PAP flap. The transverse skin paddle, 12 × 7 cm, was placed distally with the adipofascial portion positioned proximally above the muscle bellies and anastomoses site. A small raw area (4 × 3 cm) was covered with an acellular dermal matrix (ADM). The postoperative course was uneventful. At 9 months postoperatively, the patient demonstrated active flexion and extension of the fingers with independent function. The patient reported satisfaction with the flap donor site and forearm resurfacing. The PAP flap can be a further option for areas requiring soft tissue coverage in patients refusing visible scars. This flap had both the advantage of reducing the morbidity and visibility of the donor site, as well as the ability to resurface a large recipient site with soft and pliable tissue, covering exposed nerves and tendons.

## 1 INTRODUCTION

The profunda femoris artery perforator (PAP) flap has been recently popularized as an alternative option for microsurgical reconstruction (Atzeni et al., 2021; Cho et al., 2021; Ciudad et al., 2022; Heredero et al., 2021; Jo et al., 2022; Karakawa, Yoshimatsu, Fuse, et al., 2020; Kehrer et al., 2018; Martinez et al., 2021; Murphy et al., 2021). Historically, based on proximal musculocutaneous perforators from the adductor magnus (AM) muscle, it was initially referred as “adductor flap” by Angrigiani et al. (2001). Later, further publications have then better elucidated PAP's anatomy and identified clinical applications (Ahmadzadeh et al., 2007; Allen et al., 2012, 2016; Artz et al., 2021; Haddock et al., 2012; Hammond et al., 2021; Saad et al., 2012; Scaglioni et al., 2015; Song et al., 2020; Wong et al., 2015).

Soft tissue reconstruction in the forearm is often a challenging problem due to the difficulty in restoring a thin and pliable skin. While local or regional flaps are indicated for minor defects, free and distant flaps are often necessary for larger areas. Knaus et al. (2016) identified the anterolateral (ALT) free flap as one of the most useful options for forearm reconstruction because of its long pedicle, versatility and thinning (Adani et al., 2005) properties. However, the donor site is very visible and disfiguring, particularly for female patients. The superficial circumflex iliac artery perforator flap (SCIP) is also a described option for upper limb reconstruction (Berner et al., 2020), but compared to the PAP the pedicle is short and with smaller caliber of vessels; moreover, the donor site scar is more visible. Ono et al. (2017), proposed the medial plantar and the dorsalis pedis flap which can still produce unpleasant scars in the donor site with limited dimensions. Different authors (De la Garza et al., 2017; Saint Cyr & Langstein, 2006) have listed several options, such as DIEP, contralateral radial forearm, lateral arm fascia or other muscular, and fasciocutaneous free flaps. Fascial flaps have the advantage of being thin and pliable, reducing adhesions and helping underlying tendons to glide. However, these flaps leave visible donor sites and fascial flaps require skin graft application onto the fascia, which means cosmetically unsightly results at the recipient site. A recent work by Wagner et al. (2020) has reviewed the options of pedicled distant flaps from the abdominal region, that is, SCIP, SIEA, and DIEP. Although these alternatives can be useful when microsurgical reconstruction is contraindicated, they entail many disadvantages, including restriction of mobility, multiple procedures, patient discomfort, bulkiness, and visible scars.

The PAP flap can be considered a valid option because of the concealed donor site scar, a long and good caliber‐pedicle and dimensions. The profunda femoris artery runs through the posterior region of the thigh and gives off three main perforating vessels. The first perforating branch supplies the AM and gracilis muscle. The following two branches supply the biceps femoris, the semimembranosus and vastus lateralis muscles (Ahmadzadeh et al., 2007; Saad et al., 2012). At the authors' institution, the skin paddle of the flap is oriented transversally, running up to the mid‐line of the gluteal fold, irrespective of the preoperative localization of perforators, leaving a hidden scar.

According to the literature, the PAP flap has never been applied and described for reconstruction of the upper extremities, in particular the forearm. With this communication, authors intend to report the PAP flap as a further option for resurfacing defects in the upper limb.

## 2 CASE REPORT

**FIGURE 1:**
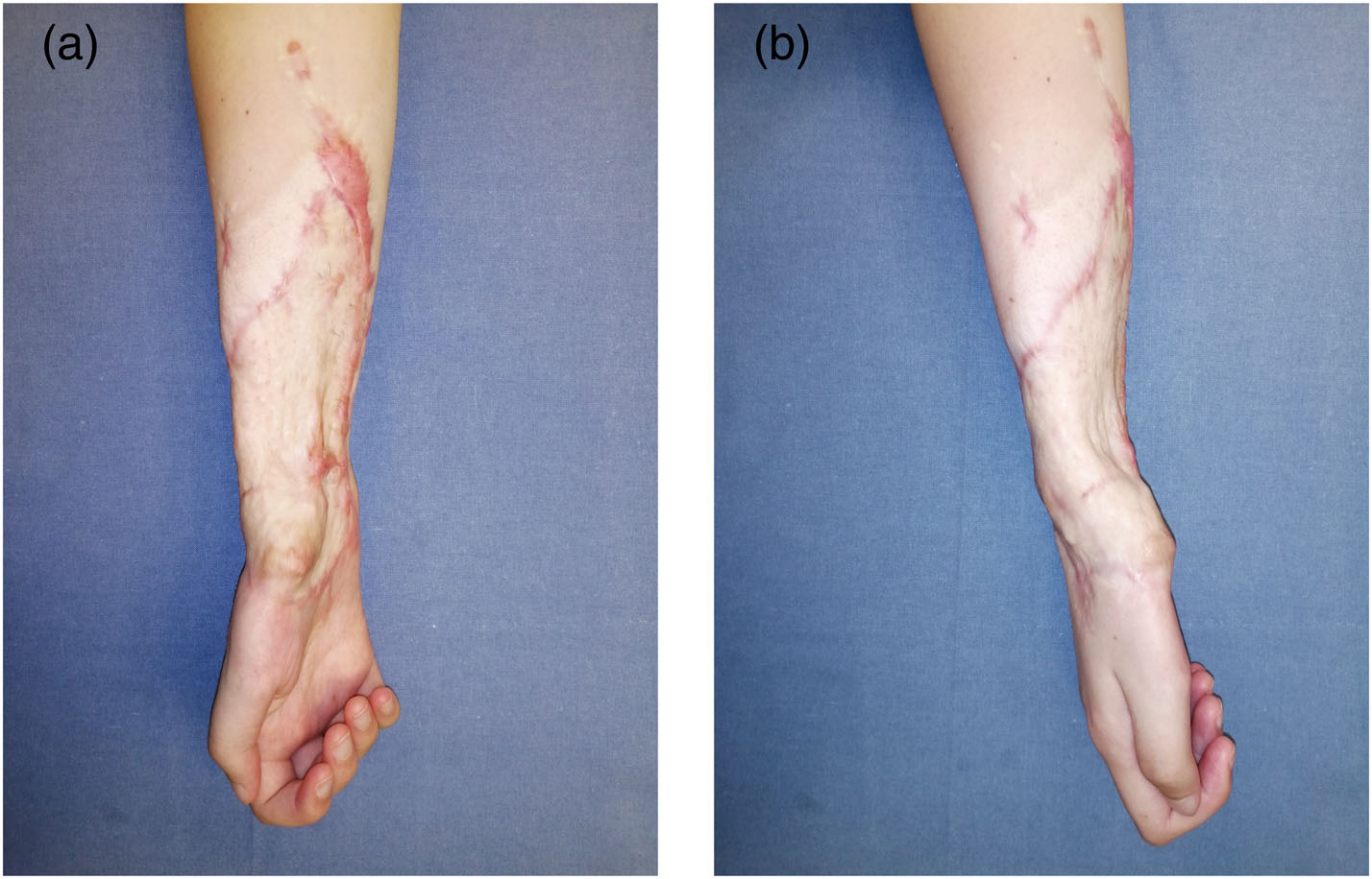
(a, b) 19‐year‐old girl with a fibrotic, adherent, and retracting scar of the right volar forearm, interfering with flexor tendon gliding. Anteroposterior (a) and lateral view (b)

**FIGURE 2:**
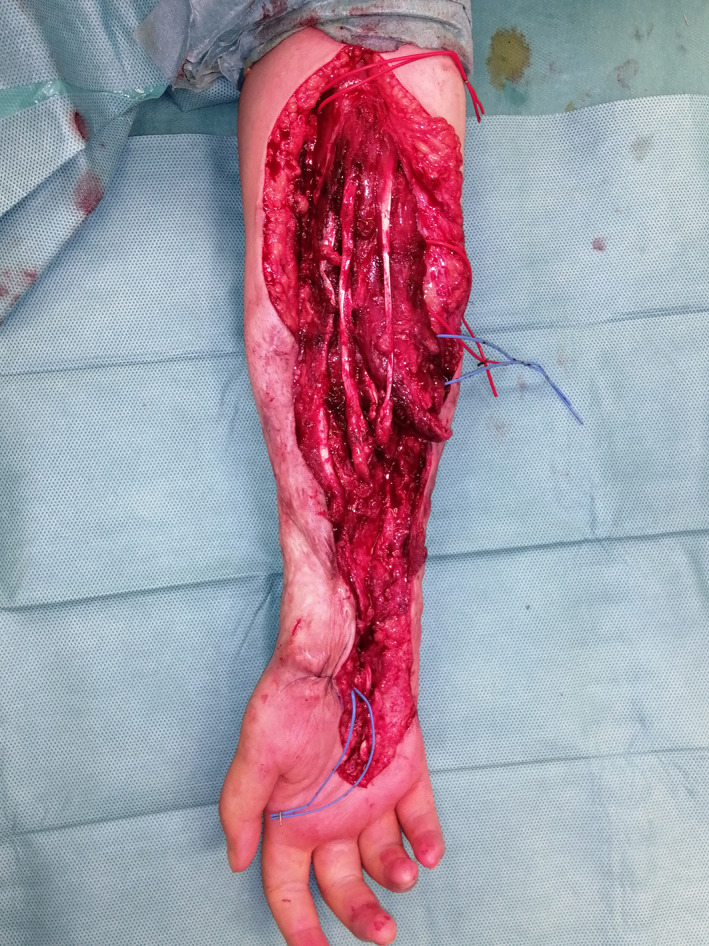
Intraoperative view after excision of the fibrotic tissue

**FIGURE 3:**
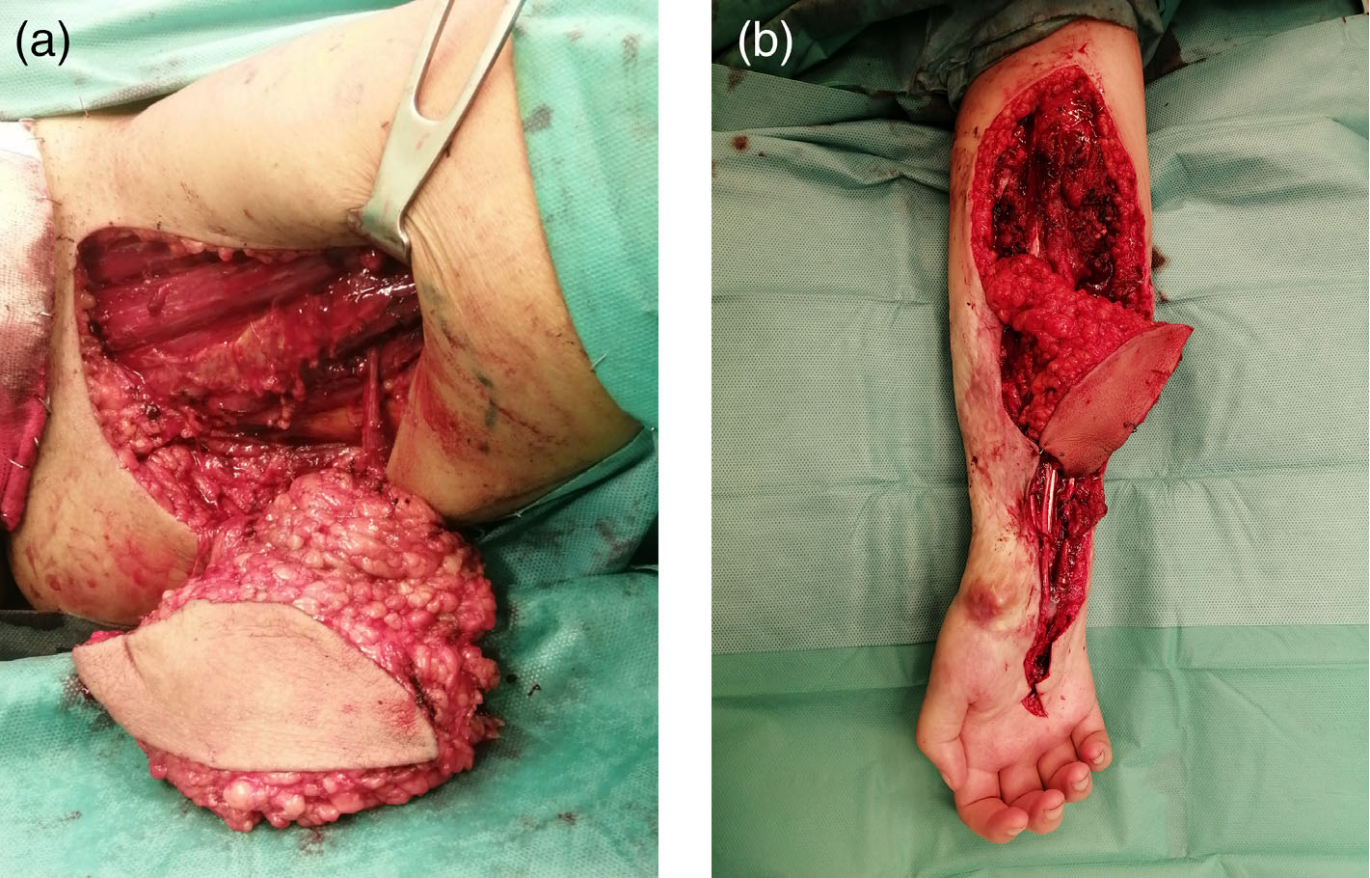
(a, b) PAP flap before vessel division and its insetting into the recipient site

**FIGURE 4:**
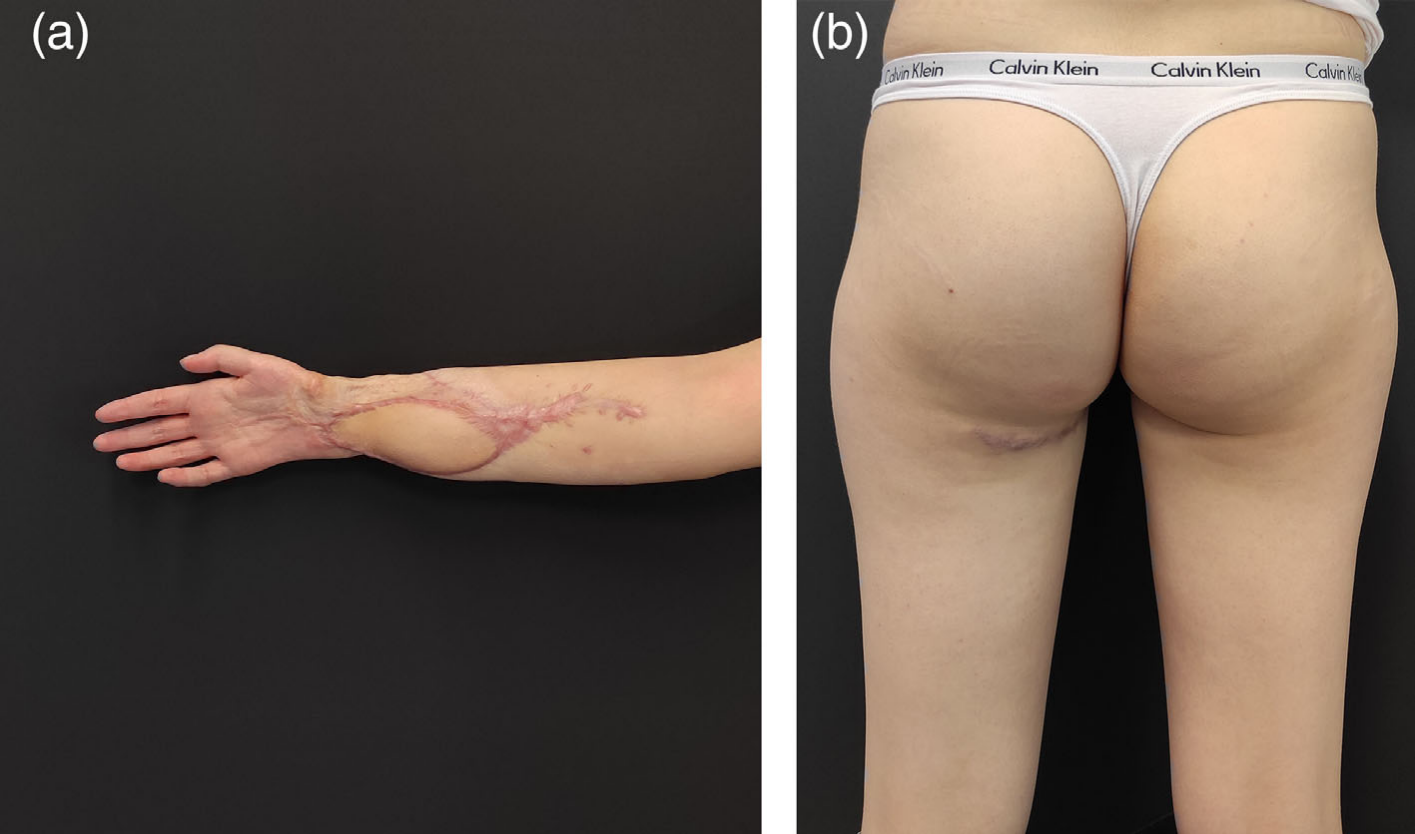
(a, b) Postoperative healing at 9 months with the recipient site (a) reacquiring flexion and extension of tendons due to the PAP flap resurfacing and the donor site (b)

A 16‐year‐old girl was seen in June 2021 with a severe flexion contracture of all fingers of the right hand, with no sensation in the tip of the fingers and thumb, with no active flexion of the fingers and wrist. A large hypertrophic scar, deeply adherent to the underlying structures, covered the entire distal half of the forearm making passive extension of the wrist and fingers impossible (Figure 1a,b). Two years earlier, the patient had a traumatic partial amputation of the forearm as a consequence of a traffic accident. At that time, an 8 cm gap in all flexor tendons and in the median nerve was documented. The ulnar nerve could not be identified distally to Guyon's canal. Radial and ulnar arteries were divided but no vascular repairs were performed. Two years after the injury, debridement, exploration with tendons and nerve repair together with soft tissue covering was offered. The patient accepted the surgical planning and among the different options for soft tissue covering: ALT, SCIP, scapular, DIEP, and PAP flaps, the patient chose the last one because of a more hidden donor site scar. The patient underwent surgical excision of the hyperthrophic contracted skin. During exploration, the palmaris longus tendon (self‐reflected) was used as an interpolated graft to the flexor pollicis longus; the flexor carpi radialis was harvested as a graft to the digitorum profundus tendons of the three ulnar digits and the brachioradialis was grafted into flexor digitorum profundus to index. The Pulvertaft technique for tendon grafting was performed. The nonfunctioning ulnar nerve was harvested and treated as an autologous bank for replacing the defect on the median nerve, through proximal and distal microsurgical coaptations (Figure 2). For the purpose of superficial soft tissue microvascular reconstruction, the radial artery stump was prepared at 6 cm distally from the antecubital fold. A left side PAP flap was prepared based on a perforating pedicle previously identified by CT angiography. The skin paddle, elliptical in shape, measured 12 × 7 cm. The perforator emerged 7 cm distally from the gluteal crease, within the undermined adipofascial part of the flap. The flap was positioned at the recipient site with the adipofascial portion covering the muscle bellies and the anastomotic site in the proximal forearm while the skin paddle resurfaced distally the scarred forearm (Figure 3a,b). Venous anastomosis was performed between the flap and the right cephalic vein, with a 2.5 mm coupler. Arterial anastomosis was performed between the PAP artery and the proximal radial artery stump with Ethilon 9/0. The proximal row area was resurfaced with ADM. At 9 months postoperatively, the volar surface of the forearm appeared soft and pliable, with no scar adhesions affecting the underlying tendon gliding Video (S1). All fingers regained active range of movement with flexion and extension and the donor site healed with no visible deformity or asymmetry between the lower limbs (Figure 4a,b).

This was confirmed by the 27 score from the validated patient reported outcome measure Body‐Q^TM^ (Klassen et al., 2016) administered at 9 months.

## 3 DISCUSSION

Originally the PAP flap was described as a pedicled option for ischiatic pressure sores (Arquette et al., 2022; Homma et al., 2001; Kim et al., 2014; Lee et al., 2009; Sharp et al., 2021) and as a free flap for head and neck reconstruction, including tongue (Heredero et al., 2021; Riera et al., 2017; Scaglioni et al., 2015). A recent expanding use is for breast reconstruction (Ahmadzadeh et al., 2007; Allen et al., 2012, 2016; Angrigiani et al., 2001; Atzeni et al., 2021; Cho et al., 2021; Jo et al., 2022; Martinez et al., 2021; Murphy et al., 2021; Saad et al., 2012; Song et al., 2020; Yano et al., 2020). Murphy et al. (2021) demonstrated the superiority of the PAP flap compared to the IGAP flap as alternative option to the DIEP flap in breast reconstruction. According to recent publications (Atzeni et al., 2021), the rate of donor site complications in PAP flap reconstruction is low and these include seroma (2.6%), wound dehiscence (2.6%), and infection (0%). Recently, the PAP flap has been described as an option for lower limb reconstruction both as a pedicled flap by Karakawa, Yoshimatsu, Maeda, et al. (2020) and as a free transfer by Ciudad et al. (2022). Another application is perineal reconstruction as described by Chang et al. (2016) and Huang et al. (2015) who report their experience in vulvar reconstruction with pedicled PAP flap. Therefore, the spectrum of utilization of PAP flap is expanding due to its reliability and versatility. Tables 1 and 2 summarize the papers describing mammary and extra‐mammary applications of PAP flap in the literature. When a free flap reconstruction is indicated, there is a growing demand for a hidden donor site scar, particularly in young female patients. The PAP flap has a constant vascular anatomy and leaves an excellent cosmesis at the donor site, since patients report that they cannot see the scar in the groin, in front of the mirror, even completely undressed. In addition, the PAP flap has a more malleable fat texture than other options. For this reason, it is an excellent option for covering areas requiring a cushioning effect or flap thinning (Heredero et al., 2020), as is the case in the forearm. Prevention of scar adhesions is of paramount importance. The technical refinements over the years have allowed sculpting of a flap consisting of a skin paddle and an adipofascial component including the main perforating pedicle. The extension of the “buried” adipofascial portion is far wider than the cutaneous part, with an “iceberg” effect, in which the tip of the iceberg overlies a far broader and thicker fatty base. This has both the advantages of reducing the morbidity of the donor site because there is less closure tension, and of sculpting a flap that can cover a large recipient site with a soft and pliable tissue, which provides a malleable and gliding tissue around nerves and tendons. In the present case, the PAP flap efficiently replaced the scar contracture and allowed an excellent tendon gliding with an early active rehabilitation protocol.

**TABLE 1:** Nonmammary applications of PAP flap

Study	Geographical origin	Number of patients	Number of flaps	Pedicled/free	Site of reconstruction	Partial/total loss	Complications
Angrigiani et al. (2001)	Argentina, USA	25	25	Pedicled (14) and free (11)	Ischial and perineal regions. Head and neck and lower limbs (free)	1/1	Not stated
Homma et al. (2001)	Japan	10	10	Pedicled	Ischial pressure ulcers	2/0	Ulcer recurrence (2)
Lee et al. (2009)	Taiwan	8	32	Pedicled (combined with other local flaps)	Ischial pressure ulcers	0/0	Ulcer recurrence (1)
Kim et al. (2014)	Korea	14	14	Pedicled (combined with gracilis/biceps flap)	Ischial pressure ulcers	0/0	Wound dehiscence (4) Congestion (1) Ulcer recurrence (1)
Sharp et al. (2021)	UK	6	18	Pedicled (combined with bilateral gracilis flap)	Pelvic reconstruction	1/0	Wound dehiscence (4) Hematoma (1)
Arquette et al. (2022)	USA	15	15	Pedicled	Perineal reconstruction	0/0	Wound dehiscence at recipient (3) Wound dehiscence at donor (3)
Fernandez Riera et al. (2017)	Taiwan	21	21	Free	Tongue	0/0	Hematoma (1) Prolonged intubation (1)
Ciudad et al. (2022)	Peru	9	9	Free	Lower limbs	1/0	Reexploration (1)
Scaglioni et al. (2015)	Taiwan	23	23	Free	Head and Neck	0/1	Pedicle thrombosis (1) Infection at recipient (1) Flap dehiscence (1) Infection at donor (2)
Karakawa, Yoshimatsu, Maeda, et al. (2020)	Japan	7	7	Pedicled	Lower limbs	0/0	Dehiscence at donor (1)
Chang et al. (2016)	Taiwan	12	19	Pedicled	Vulva	0/0	Poor wound healing (7) Infection (3) Hematoma (2) Flap revision (5)
Huang et al. (2015)	Taiwan	16	11	Pedicled	Vulva	0/0	Wound poor healing (6) Infection (3) Hematoma (1) Flap revision (1)
Heredero et al. (2020)	Spain, USA	10	10	Free	Tongue	0/1	Vasospasm followed by flap failure (1)
Largo et al. (2020)	USA	60	61	Free	Head and Neck (majority)	0/0	Wound dehiscence (8) Arterial flap thrombosis (1) surgically revised
Ito et al. (2016)	Taiwan	48	48	Free	Head and Neck	3/0	Pedicle thrombosis (3)

**TABLE 2:** Mammary applications of PAP flap

Study	Geographical origin	Number of patients	Number of flaps	Partial/total loss	Complications
Jo et al. (2022)	Korea	32	43	1/0	Venous congestion (1) Wound dehiscence at donor (2) Fat necrosis (1)
Atzeni et al. (2021)	Italy	86	116	0/0	Seroma (3) Hematoma (2) Wound dehiscence (3) Fat necrosis (2)
Tielemans et al. (2021)	Netherland	28	46	1/0	Wound dehiscence at recipient (1) Wound dehiscence at donor (1)
Hunsinger et al. (2019)	France	51	62	0/2	Seroma (3) Wound dehiscence (7) Pedicle thrombosis (2)
Haddock et al. (2020)	USA	138	265	0/8	Seroma (12) Hematoma (3) Wound dehiscence (18)
Haddad et al. (2016)	France	25	30	0/2	Seroma (2) Hematoma (1) Wound dehiscence (4) Pedicle thrombosis (2)
Hupkens et al. (2016)	Netherland	30	40	0/0	Wound dehiscence (4)
Fosseprez et al. (2017)	Belgium	15	17	0/2	Hematoma (1) Wound dehiscence (7) Fat necrosis (1)
Allen et al. (2012)	USA	15	27	2/0	Seroma (1) Hematoma (1)
Allen et al. (2016)	USA	96	164	0/1	Seroma (10) Hematoma (3) Wound dehiscence (6) Fat necrosis (11)

The donor scar healed without complication and the Body‐Q score confirmed the satisfactory acceptance of the donor scar in a young and sportive woman, without functional impairment (Haddock & Teotia, 2020).

This flap does have limitations. The main one is the potential bulky nature related to the adipose accumulation characterizing the inner thigh. However, this feature is variable among the general population, and in the specific case, the patient was thin and athletic, therefore, the amount of subcutaneous fat tissue was not significantly greater than other reconstructive options, that is, the super thin ALT or the extended SCIP. Furthermore, this flap was specifically requested by the patient because of the hidden donor scar compared to the other flap options. In addition to this, the particular construct of the flap as described before, allows the advantages of both adipofascial flaps and skin paddle‐bearing flaps. In fact, the adipofascial portion is made of loose elastic areolar tissue, which can be thinned, stretched, customized and adapted even to areas requiring thin reconstructions such as the upper limb. The possibility of customizing the extension and thickness of the PAP flap has been mentioned in the medical literature, respectively, by Tialemans et al. (2021) and Heredero et al. (2020) who defines the thinning of this flap with high BMI patients for the purpose of tongue reconstruction. A longer case series is necessary to make meaningful conclusions about the use of the PAP flap for forearm reconstruction but this case report describes this flap as an available option.

## Supporting information


**VIDEO S1**: Flexion and extension of the right hand fingers at 6 months postoperatively.

## Data Availability

The data that support the findings of this study are available on request from the corresponding author. The data are not publicly available due to privacy or ethical restrictions.
